# How to avoid complications of distraction osteogenesis for first brachymetatarsia

**DOI:** 10.3109/17453670902930040

**Published:** 2009-04-01

**Authors:** Keun-Bae Lee, Hyun-Kee Yang, Jae-Yoon Chung, Eun-Sun Moon, Sung-Taek Jung

**Affiliations:** Department of Orthopaedics, Chonnam National University Medical School and HospitalGwangjuSouth Korea

## Abstract

**Background and purpose** Distraction osteogenesis may be used for the treatment of brachymetatarsia. However, few reports have been published on first metatarsal lengthening by this method. We evaluated the complications of distraction osteogenesis for first brachymetatarsia and here we provide a solution.

**Patients and methods** 16 patients (27 feet) underwent distraction osteogenesis for first brachymetatarsia. Mean age at time of surgery was 20 (12–34) years and mean duration of postoperative follow-up was 5 (2–13) years. A unilateral external fixator was fixed at the medial aspect of the metatarsus. The distraction axis was parallel to the plantar surface of the foot in the sagittal plane and to the second metatarsal axis in the transversal plane.

**Results** First metatarsal length expressed as a proportion of second metatarsal length was 60% (55–64). Average degree of metatarsal lengthening was 42% (34–54), and the average lengthening index was 64 (39–93) days/cm. The most common complication was stiffness of the metatarsophalangeal joint (12 feet). Deformities that included cavus foot and hallux valgus occurred in 3 feet each, and callus fractures occurred in 3 feet. The other complications were pin breakage and pin tract infection in 2 feet each.

**Interpretation** Distraction osteogenesis for first brachymetatarsia can give satisfactory cosmetic and functional results. However, several complications are commonly encountered. This report on complications and their solutions may help those attempting distraction osteogenesis for first brachymetatarsia.

## Introduction

Brachymetatarsia is a congenital deformity and involves an abnormally short metatarsal caused by premature closure of the epiphysis. The fourth toe is most commonly involved, followed by the first and the fifth toes (Viladot 1973). The deformity is not only cosmetically disturbing but also impairs the weight-bearing mechanism of the foot. In first brachymetatarsia, excessive weight bearing is loaded onto the second and third metatarsus by a change in weight, and pressure and pain can develop during shoe wearing and walking because of the deformity and overlapping adjacent toes (Tanaka et al. 1995, Baek and Chung 1998). Surgery should be planned on the basis of functional recovery and anatomical restoration that restores proper weight bearing in addition to addressing cosmetic problems. Several techniques have been described for the treatment of brachymetatarsia; these include one-stage lengthening using an interpositional bone graft, gradual lengthening by distraction osteogenesis with or without shortening of the adjacent metatarsals and phalanges, and shortening of adjacent metatarsals and phalanges (Harris and Beath 1949, Hughes et al. 1990, Kawashima et al. 1994, Choi et al. 1999, Masada et al. 1999, Oh et al. 2004).

The most widely used procedures are one-stage lengthening or distraction osteogenesis. Each method has its own advantages and disadvantages. The advantages of one-stage lengthening include a shorter period of bony union, better patient compliance, and less scar formation. However, this method has the disadvantages of donor site morbidity, neurovascular impairment, and smaller length gain (Harris and Beath 1949, Hughes et al. 1990). Congenital shortness of the metatarsal bone represents one of the best indications for distraction osteogenesis in the foot (Martin 2001). The advantages of distraction osteogenesis include: no need for bone grafting, easier tendon stretching, fewer neurovascular complications, early weight bearing, and a larger length gain, whereas disadvantages include stiffness or subluxation of the metatarsophalangeal (MTP) joint, cavus or angulation deformity, pin tract infection, and a longer period of bony union (Kawashima et al. 1994, Takakura et al. 1997, Choi et al. 1999, Masada et al. 1999, Oh et al. 2004, Shim and Park 2006). However, few reports have been published on first metatarsal lengthening by this method (Takakura et al. 1997, Oh et al. 2004, Kim et al. 2003, Shim and Park 2006). We evaluated the results and complications that occurred in 27 feet with brachymetatarsia of the first ray treated with distraction osteogenesis, and here we provide solutions for complications.

## Patients and methods

16 patients (15 women) underwent distraction osteogenesis for first brachymetatarsia from January, 1998 to June, 2006 at our institution (27 feet). Metatarsal shortening was congenital in all patients. The mean age at time of surgery was 20 (12–34) years and mean duration of postoperative follow-up was 62 (26–126) months. All the patients except 3 (nos. 1, 2, and 8) were treated after epiphyseal closure of the metatarsus. Before surgery, we made patients and parents aware of the various complications associated with distraction osteogenesis (e.g. loss of motion, sensitivity about the scar). Surgery was aimed at functional recovery and anatomical restoration to recover proper weight bearing and to minimize cosmetic problems. 11 patients underwent bilateral first metatarsal lengthening, and 9 patients (13 feet) underwent simultaneous lengthening of the fourth metatarsal bone. 14 of the 16 patients had occasional pain around adjacent toes when wearing shoes and walking. 2 other patients complained of psychological distress caused by the cosmetic appearance of their deformity.

### Operative technique

**Figure 1.**
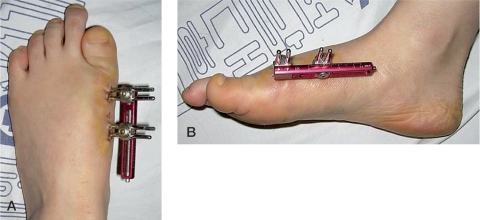
The surgical technique to prevent metatarsal deformity during distraction osteogenesis for first brachymetatarsia. A. The axis of the external fixator must be aligned parallel to the anatomical axis of the second metatarsal in the transversal plane. 4 miniature half-pins were inserted into the proximal and distal metaphysis (2 into each) and directed medial to lateral. B. The axis of the external fixator should be as parallel as possible to the plantar surface at the medial aspect of the first metatarsal bone.

Under fluoroscopic guidance, 2 miniature half-pins were inserted into the proximal metaphysis and 2 into the distal metaphysis, and directed medial to lateral. When the first metatarsal bone was too short to accept all 4 half-pins, 1 proximal pin was inserted in the medial cuneiform. A unilateral external fixator (Dyna-EXTOR, BK meditech, Seoul, Korea) was fixed at the medial aspect of the metatarsus, as parallel to the plantar surface as possible in the sagittal plane, and as parallel to the second metatarsal axis in the transversal plane to prevent malalignment during metatarsal lengthening (Figure 1). A medial longitudinal incision of 1.5 cm was made at the metatarsal shaft and corticotomy was performed perpendicular to the plantar surface of the foot. We did not perform plantar fascia release or flexor tenotomy to lengthen soft tissue. The proper direction of the metatarsal lengthening was confirmed by a predistraction of about 5 mm by intraoperative fluoroscanning.

Postoperatively, 0.25 mm of distraction was applied 3 times a day after a 7-day latent period. Radiographs were checked every other week to inspect the degree of osteogenesis and joint condition. We controlled distraction rate and rhythm according to radiographic findings and the clinical condition of the patient. Distraction was continued until a satisfactory metatarsal length had been achieved. Patients were permitted full weight-bearing ambulation with heel touching using postoperative shoes on the second postoperative day. The external fixators were removed when calluses had matured.

### Evaluation of radiographic and clinical outcome

Radiographs were taken with the foot plantigrade on the floor, and the radiation beam was directed at the center of the forefoot and was inclined 15 degrees from the vertical at a distance of 100 cm above the film to obtain anteroposterior radiographs. We evaluated first metatarsal lengths as percentages of second metatarsal lengths, lengthening gains and percentages, external fixation periods, lengthening index, and ranges of motion of first MTP joints. First and second metatarsal lengths were measured according to Tanaka et al. (1995). Briefly, the axes of the first and second metatarsals were lines that connected the mid-points of the proximal and distal ends of their diaphysis. Metatarsal length is the distance between the points of intersection of the axes of the metatarsals with the distal ends and proximal ends of the metatarsals.

External fixation period was from the date of surgery to fixator removal. Lengthening index was calculated by dividing the length of the external fixation period by the length gained. Patients were allocated to 3 groups with respect to MTP joint motion (dorsiflexion plus plantarflexion), i.e. normal or mild restriction (≥ 75˚), moderate restriction (30˚–74˚), or marked restriction (< 30˚). We defined stiffness as being moderate or marked restriction of MTP joint motion. The outcome was assessed clinically according to the American Orthopaedic Foot and Ankle Society (AOFAS) hallux metatarsophalangeal-interphalangeal scale (Kitaoka et al. 1994) and graded as excellent (> 85), good (71–85), fair (56–70), or poor (< 56).

## Results

Mean length of the first metatarsal expressed as a proportion of that of the second metatarsal was 60% (55–64). Average length gained was 17 (13–21) mm, and average length gained as a proportion of the original metatarsal length was 42% (34–54). Bony consolidation was achieved in all 27 metatarsals. Average external fixation time was 108 (68–132) days, and mean lengthening index was 64 (39–93) days/cm. The mean MTP joint range of motion preoperatively was 58 (50–65) and 32 (25–35) degrees for dorsi- and plantar-flexion, respectively, and 45 (25–65) and 26 (20–35) degrees at the last follow-up. MTP joint motion showed normal or mild restriction in 15 feet, and moderate restriction in 12 feet (Table 1).

**Table 1.** Details of 27 feet in 16 patients with first brachymetatarsia who underwent distraction osteogenesis

A	B	C	D	E	F	G		H	I	J		K	L	
						a	b			a	b		a	b
1	12	F	R	4th	59	19	49	122	64	65/30	40/20	Hallux valgus	87	90
			L	None	60	18	44	77	43	65/30	55/30	None	90	97
2	13	F	R	4th	62	17	39	90	53	55/25	40/20	Hallux valgus	85	90
			L	4th	62	17	39	68	40	55/25	40/20	Pin infection	85	95
3	22	F	R	None	61	17	39	106	62	60/30	50/30	None	90	100
			L	None	61	17	38	81	48	50/30	40/20	Pin infection	87	95
4	15	F	L	4th	57	19	54	121	64	65/35	65/30	None	90	97
5	21	F	L	4th	59	17	39	106	62	60/35	50/30	None	90	100
6	13	F	R	4th	61	19	54	74	39	60/30	50/20	Hallux valgus	85	80
			L	4th	61	19	52	74	39	60/30	40/20	None	85	95
7	17	F	R	4th	56	21	51	115	55	55/30	40/20	Pin breakage	85	85
			L	None	59	19	47	115	61	65/35	55/35	None	90	100
8	13	F	L	4th	62	19	51	114	60	60/35	50/35	None	90	100
9	27	F	R	4th	61	20	51	122	61	55/30	30/25	Cavus deformity	85	85
			L	4th	60	15	37	122	81	55/30	25/25	Cavus deformity	85	80
10	20	M	R	None	60	18	38	132	73	55/30	40/20	None	85	90
			L	None	60	16	34	132	83	55/30	35/20	Cavus deformity		
												Pin breakage	85	80
11	33	F	R	4th	62	14	37	130	93	60/30	50/25	Callus fracture	85	80
			L	4th	59	13	35	115	88	60/30	50/25	None	85	85
12	25	F	R	None	62	15	37	97	65	60/35	50/30	None	90	100
			L	None	60	15	34	97	65	60/35	50/30	None	90	97
13	34	F	R	None	64	18	43	125	69	60/35	50/30	Callus fracture	85	90
			L	None	62	17	41	125	74	60/35	50/30	Callus fracture	87	90
14	18	F	L	None	60	14	37	125	89	55/35	50/35	None	90	95
15	14	F	R	None	55	15	38	90	60	50/35	35/25	None	90	85
			L	None	56	15	35	131	87	55/35	45/30	None	90	97
16	24	F	R	None	62	18	38	113	63	55/35	45/30	None	90	100
Mean	20				60	17	42	108	64	58/32	45/26		87	92

MT1: first metatarsal; MT2: second metatarsal; DF, dorsiflexion; PF: plantar flexion; F/U: follow-up.

AOFAS: American Orthopaedic Foot and Ankle Society hallux metatarsophalangeal-interphalangeal scale (excellent, > 85; good, 85–71; fair, 70–56; and poor, < 56)

A Pat. No.

B Age (yr)

C Sex

D Side

E Other metatarsal lengthening

F Initial length of MT1 as % of MT2

G MT1 length gain a. mm b. %

H External fixator time

I Lengthening Index (Days/cm)

J MTP joint ROM (DF/PF) a. Preop. b. Last F/U

K Complications

L Results (AOFAS score) a. Preop. b. Last F/U

**Figure 2.**
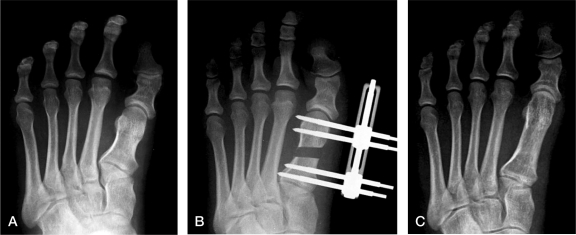
A. Preoperatively: shortening of the first metatarsal. B. Patient undergoing lengthening of the first metatarsal by distraction osteogenesis. C. Excellent final radiographic result.

The mean AOFAS score was 87 (85–90) points preoperatively and improved to 92 (80–100) points at the last follow-up; 19 cases had excellent results (Figure 2) and 8 had good results. 3 patients whose epiphysis of the metatarsus was not fused before surgery had no problems associated with residual growth at the last follow-up. The 3 patients (nos. 14, 15, and 16) with the shortest follow-up after external fixator removal (22, 23, and 22 months) had no complications.

### Complications

**Figure 3.**
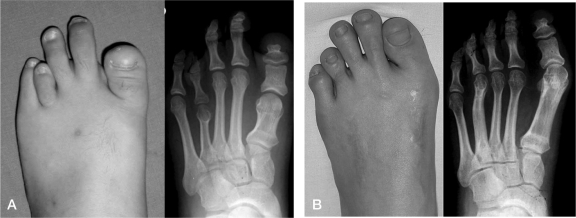
A. Preoperatively: shortening of the first and fourth metatarsals. The patient underwent simultaneous lengthening of the first and fourth metatarsal bones by distraction osteogenesis. B. After consolidation, with lengthened first metatarsals and moderate hallux valgus deformity. The patient accepted the results because of the cosmetic improvement and absence of bunion pain.

**Figure 4.**
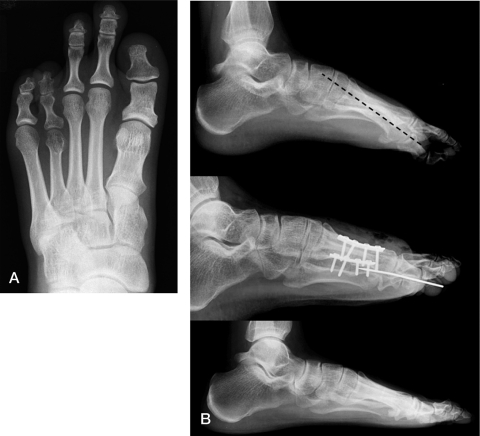
A. Shortening of the first and fourth metatarsals. B. Lateral radiography after consolidation of lengthened metatarsal bone showed cavus deformities (top, dotted line) of the first and fourth metatarsals. Dorsal closing wedge osteotomies were performed (middle) and the final radiograph (bottom) shows correction of cavus deformity.

The most common complication encountered was MTP joint stiffness, which was seen in 12 feet. 3 patients (3 feet) had moderate hallux valgus deformity. 2 of these patients accepted their results because of cosmetic improvement and no bunion pain (Figure 3). 1 patient developed painful hallux valgus, which was later corrected by distal chevron osteotomy. Cavus deformity of the metatarsus occurred in 2 patients who had no cavus deformity before surgery (3 feet). 1 patient with bilateral deformity underwent dorsal closing wedge osteotomies at the metatarsal base and correction was achieved (Figure 4). Another patient accepted the deformity cosmetically and functionally. All 3 feet were plantigrade without any symptoms at final follow-up. Callus fractures occurred in two patients who underwent simultaneous distraction osteogenesis for bilateral first brachymetatarsia (3 feet). They were converted from external fixation to internal fixation with a plate, and then achieved bony consolidation. 2 pin breakages that occurred during the lengthening period in 2 patients were treated by changing of pins, and 2 cases of pin tract infection were uneventfully resolved by oral antibiotics and dressing. We did not encounter MTP joint subluxation, MTP joint arthrosis, or neurovascular complications.

## Discussion

Various complications of distraction osteogenesis, as now generally performed to correct first brachymetatarsia have been reported, e.g. stiffness or subluxation of the MTP joint, cavus or other angulation deformity of the metatarsus, prolonged time for bony consolidation, and pin tract infection (Table 2) (Kawashima et al. 1994, Takakura et al. 1997, Masada et al 1999, Oh et al. 2004).

**Table 2.** Summary of literature on complications after distraction osteogenesis for first brachymetatarsia

Study	No. of cases	Average age at surgery (years)	Gain in length (%)	Mean lengthening index (days/cm)	Follow-up (months)	Complications								Total no. of cases with complications
						A	B	C	D	E	F	G	H	
Takakura et al. (1997)	7	22	31	75	53	1	0	0	0	0	7	0	0	7
Kim et al. (2003)	10	14	52	61	36	1	0	1	0	0	1	0	0	3
Oh et al. (2004)	13	19	49	60	27	2	4	0	1	1	7	0	2	9
Current study	27	20	39	64	62	3	3	3	0	0	12	2	2	15

A: Hallus valgus; B: cavus deformity; C: callus fracture; D: medial angulation; E: lateral angulation; F: metatarsophalangeal joint stiffness; G: pin breakage; H: pin infection.

The most common complication is MTP joint stiffness. Although loss of any motion or loss of more than 50% of dorsiflexion has been considered to be MTP joint stiffness in several studies (Takakura et al. 1997, Masada et al 1999, Shim and Park 2006), we considered MTP joint motion of below 75 degrees to be stiffness. In healthy individuals, the average length of the first metatarsal expressed as a proportion of of the length of the second metatarsal is about 86% (Kitaoka et al. 1994). Moreover, a few authors (Takakura et al. 1997, Masada et al 1999, Shim and Park 2006) have reported greater reduction in motion in patients with a length gain of more than 40%, and have emphasized that the amount of lengthening should not exceed 40%. To avoid excessive lengthening, Kim et al. (2003) recommended shortening of an adjacent bone for the treatment of first brachymetatarsia when the amount of lengthening is expected to exceed 40%. Also, Kawashima et al. (1994) reported that a distraction rate of 0.35 mm twice a day is too fast for tendon elongation, although adequate for bone distraction. Choi et al. (1999) reported that a distraction rate of 0.25 mm twice a day is optimal for soft tissue elongation and bone formation. In our study, distraction was performed at 0.25 mm 3 times a day and MTP joint stiffness was observed in 13 of the 27 feet. Based on our results, MTP joint stiffness may be caused by an excessive amount of lengthening, a distraction rate and rhythm that are too rapid, or a long period of external fixation. The best prevention against joint stiffness is stretching therapy, if it can be instituted safety (Martin 2001). If therapy is unable to restore adequate motion, then tendon lengthening may be necessary (Martin 2001). Shim and Park (2006) reported improvement of 7 cases who underwent plantar capsular release on flexion contracture or subluxation of the MTP joint after distraction osteogenesis. Thus, we consider that the amount of lengthening should be less than 40%, and that a distraction rate of 0.25 mm twice a day is optimal; and furthermore, that postoperative active and passive MTP joint mobilization exercises are essential for preventing MTP joint stiffness.

Malalignments after distraction osteogenesis, such as cavus or hallux valgus deformities, are also important complications. Oh et al. (2004) reported 2 cases of cavus deformity due to tight plantar soft tissues in 2 patients who had undergone considerable lengthenings (56% and 66%). The other factor that favors cavus deformity concerns the anatomical orientation of the first metatarsal (Oh et al. 2004). Normally, the first metatarsal has approximately 30 degrees of inclination in the sagittal plane. If lengthening is performed along the anatomical axis of the first metatarsal, the head of the first metatarsal will be situated at a lower position than the plantar surface in the final lengthened state. Thus, in an attempt to prevent the development of cavus deformity during and after lengthening, the axis of the external fixator must be as parallel as possible to the plantar surface at the medial aspect of the first metatarsal bone. Even so, in our study, cavus deformity developed in 2 patients (3 feet). In 1 patient, the deformity was bilateral with painful plantar callosities, and the patient underwent dorsal closing wedge osteotomies at the metatarsal base. We did not perform plantar fascia release and flexor tenotomy to prevent cavus deformity during the index procedure. Thus, we believe that the soft tissue procedure will also be needed in cases with tight plantar soft tissue.

Hallux valgus deformity was caused by the anatomical orientation of the first metatarsal. If lengthening is performed along the anatomical axis of the first metatarsal with an increased intermetatarsal angle, the hallux valgus angle will be increased after final lengthening. Thus, to prevent the development of hallux valgus deformity, the axis of the external fixator must be aligned parallel to the anatomical axis of the second metatarsal in the transversal plane.

In cases treated at our institution before this study was initiated, the external fixator was attached at the dorsal aspect of the first metatarsal, and complications such as cavus deformity and angulation deformity occurred frequently. We therefore changed the external fixator position from the dorsal side to the medial aspect of the first metatarsal, and found that complications of malalignment were markedly reduced.

Callus fractures occurred in 2 patients (3 feet) who were allowed to walk with heel weight bearing as soon as they could tolerate the pain. However, they walked using the entire sole. We believe that the callus fractures may have been caused by too rapid a distraction rate and walking with weight bearing using the whole sole, which would result in overloading of the callus during ambulation. We thus advocate that full weight-bearing ambulation postoperatively without support should be delayed in patients who have undergone simultaneous correction for bilateral first brachymetatarsia.

We conclude that distraction osteogenesis for first brachymetatarsia can give satisfactory cosmetic and functional results. However, several complications are commonly encountered. We consider that these could be reduced by using the technique described above.
